# Enhancing Oral Bioavailability of Simvastatin Using Uncoated and Polymer-Coated Solid Lipid Nanoparticles

**DOI:** 10.3390/pharmaceutics16060763

**Published:** 2024-06-04

**Authors:** Amira E. Abd-Elghany, Omar El-Garhy, Adel Al Fatease, Ali H. Alamri, Hamdy Abdelkader

**Affiliations:** 1 Department of Pharmaceutics, Faculty of Pharmacy, Minia University, Minia 61519, Egypt; amiraemad211@gmail.com (A.E.A.-E.); omar.elgarhy@mu.edu.eg (O.E.-G.); 2 Department of Pharmaceutics, College of Pharmacy, King Khalid University, Abha 62223, Saudi Arabia; afatease@kku.edu.sa (A.A.F.); aamri@kku.edu.sa (A.H.A.)

**Keywords:** solid lipid nanoparticles, polymeric coating, Precirol ATO 5, Compritol 888 ATO, chitosan, simvastatin

## Abstract

Simvastatin (SVA) is a well-prescribed drug for treating cardiovascular and hypercholesterolemia. Due to the extensive hepatic first-pass metabolism and poor solubility, its oral bioavailability is 5%. Solid lipid nanoparticles (SLNs) and hydrogel-coated SLNs were investigated to overcome the limited bioavailability of SVA. Four different lipids used alone or in combination with two stabilizers were employed to generate 13 SLNs. Two concentrations of chitosan (CS) and alginate (AL) were coating materials. SLNs were studied for particle size, zeta potential, in vitro release, rheology, and bioavailability. The viscosities of both the bare and coated SLNs exhibited shear-thinning behavior. The viscosity of F11 (Chitosan 1%) at 20 and 40 rpm were 424 and 168 cp, respectively. F11 had a particle size of 260.1 ± 3.72 nm with a higher release; the particle size of F11-CS at 1% was 524.3 ± 80.31 nm. In vivo studies illustrated that F11 had the highest plasma concentration when compared with the SVA suspension and coated chitosan (F11 (Chitosan 1%)). Greater bioavailability is measured as (AUC0→24), as compared to uncoated ones. The AUC for F11, F11-CS 1%, and the SVA suspension were 1880.4, 3562.18, and 272 ng·h/mL, respectively. Both bare and coated SLNs exhibited a significantly higher relative bioavailability when compared to that from the control SVA.

## 1. Introduction

According to a WHO report released in 2021, cardiovascular diseases are the main cause of death worldwide. In 2019, a global estimate projected that approximately 18 million people died from cardiovascular diseases [1]. Hypercholesterolemia is also associated with other serious conditions. For example, the second most prevalent cause of retinal disorders, retinal vein occlusion (RVO), retinal bleeding, and swelling results from hypocholesteremia, which may progress to a partial or complete loss of vision [2,3]. All previous diseases can be treated with statins [2,3]. Statins are commonly used worldwide. Statins are HMG-CoA reductase inhibitors that lower lipid levels by inhibiting HMG-CoA reductase activity. This enzyme primarily regulates lipid production in hepatocytes [4,5]. Statins provide both cholesterol-lowering and non-cholesterol (pleiotropic) actions. They also have the essential function of preventing cardiac diseases (e.g., heart attacks and strokes) caused by high lipid levels and atherosclerotic lesions.

The statin group consists of seven different members as follows: simvastatin, lovastatin, pravastatin, fluvastatin, atorvastatin, rosuvastatin, and cerivastatin. One of the statin classes (Cerivastatin) has recently been removed from pharmacy shelves because of 52 deaths associated with drug-related myopathy and kidney damage [6,7]. Simvastatin, lovastatin, and pravastatin are derived from fungi, while fluvastatin, atorvastatin, and rosuvastatin are synthetic drugs [8]. Several statins including simvastatin (SVA) are poorly water-soluble which can lead to poor in vivo dissolution with implications for bioavailability. Secondly, some statins, such as SVA, are subject to significant first-pass metabolism which has a major impact on bioavailability.

Fewer side effects were recorded for SVA. One of the most significant disadvantages of statin drugs is their low bioavailability, which causes a minimum rate of medication dissolution in the GI tract (GIT). In addition, many statins including simvastatin undergo significant deactivation due to extensive first-pass metabolism prior to reaching the general circulation, hence causing low bioavailability [9].

The pharmacokinetics and clinical efficacy of various statin compounds can be determined based on their physicochemical properties. For example, the solubility of statins in water varies, as seen by their partition coefficients (log P), which vary from −0.25 to 4.5. The active chemicals are all acidic, with a pKa rating of approximately 5.5 [10]. Several statins, including SVA, have been marketed as immediate-release tablets. SVA has an extremely short half-life of 3 h, and it is eliminated through the extensive cytochrome P3A metabolism in the liver and intestines. SVA has a restricted oral bioavailability of only 5% in humans because of its slow rate of dissolution and significant first-pass effect. The poor solubility and high permeability lead to extensive first-pass metabolism characteristics, likely placing SVA under borderline class II/IV of the BCS [11].

The restricted bioavailability and poor water solubility of SVA have recently been overcome in several ways. Nanoparticles, size reduction, solid dispersions, cyclodextrins, niosomes, phospholipid vesicles, and other carriers have been investigated to achieve low solubility and permeability [12].

Solid lipid nanoparticles (SLNs) are spherical lipid-based particles. In contrast to phospholipid- and surfactant-based vesicles, SLNs are formed from high-melting-point lipids that are solid even under ambient and body conditions, and can also be stabilized by surfactants. The lipids may include pure triglycerides, complex glycerol mixtures, and/or waxy lipids. The average size of SLNs was small (≤1000 µm) [13]. The beneficial components of SLNs are non-toxic and biodegradable. A unique characteristic of SLNs is their capacity to transport a range of pharmaceuticals, including minor pharmacological chemicals, biomacromolecules, genetic structures (DNA and siRNA), and vaccine antigens [14].

The solid fatty core matrix of SLNs encapsulating drug molecules has been reported to release the payload in a regulated manner both in vitro and in vivo. Few experimental studies have focused on using SLNs to prevent drugs, such as atorvastatin and lovastatin from metabolizing in the first pass [15]. The results showed that the type and ratio of lipids and liquid phases significantly affected the particle size, zeta potential, and drug release profile of SLNs. When mucoadhesive polymers are used with SLNs, some beneficial features can be offered to the bare SLNs, such as preserving compounds against biological forces and enhancing oral bioavailability [16].

Chitosan is advantageous for medicinal applications because of the unique qualities of its main amine groups. Chitosan is commonly used in many industries, including the cosmetic, culinary, and agricultural sectors. Chitosan has acceptable biocompatibility, safety, and biodegradability [16]. Alginate sodium is an anionic polymer and one of the most frequently used materials for various medicinal purposes. Because of its nontoxicity, biodegradability, biocompatibility, and gelation, alginate sodium is a potential anionic polymer for drug delivery [17].

In this study, various lipids with different HLB values, such as glyceryl palmitostearate (commercially known as Precirol ATO 5), glyceryl dibehenate (Compritol 888 ATO), mono- and diglycerides (Geleol), and polyglycolic glycol esters (Gelucire 43/01), were employed to fabricate SLNs loaded with simvastatin. Two stabilizers were investigated, namely Tween 80 and Poloxamer 407.

In addition, some selected SLNs were coated with the following two mucoadhesive polymers: chitosan and sodium alginate. The prepared SLNs were evaluated for particle size, zeta potential, morphology, in vitro release, rheological properties, and bioavailability.

## 2. Materials and Methods

SVA was provided by Amrya Pharmaceuticals, Alexandria, Egypt. Precirol ATO 5 (Glyceryl palmitostearate), Geleol (mono and diglycerides), Gelucire 43/01 pellets (polyglycolic glycol esters), and Compritol 888 ATO (Glyceryl dibehenate) were provided as a gift from Gattefosse, Cedex, France. Tween 80 was purchased from Oxford Laboratory, Mumbai, India. Dialysis membranes (MWCO of 12–14 kDa), sodium alginate, chitosan, Poloxamer 407, and acetic acid (HPLC grade) were obtained from Sigma Aldrich Chemicals Co., St. Louis, MO, USA.

### 2.1. Preparation of Simvastatin-Loaded Solid Lipid Nanoparticles (SLNs)

Simvastatin-loaded SLNs were prepared using the hot homogenization method [18]. Thirteen SLNs were constructed, as shown in Table 1. The lipophilic phase is composed of lipids (e.g., Compritol 888 ATO or a mix of lipids (Compritol 888 ATO and Gelucire 43/01), as shown in Table 1. The lipid mixture was melted at 70 °C in a conical flask, and the active substance simvastatin (10 mg) was dissolved in the melted lipid(s) under magnetic stirring. Poloxamer 407 (100 mg) and polysorbate 80 (5 mg/mL) were dissolved in distilled water (10 mL) at 70 °C. Homogenization (IKA T-18 Basic Ultra-turrax, Staufen im Breisgau, Germany) was performed for three minutes (min) at 12,000 rpm under moderate magnetic stirring (250–300 rpm) to form an emulsion of the two phases. Upon cooling, milky dispersions of SLNs were formed [19].

### 2.2. Preparation of Solution of Na-Alginate/Chitosan Coating Layer at 1% and 0.5% (w/v)

Specific amounts (0.5 and 1 g) of sodium alginate were dissolved separately in 100 mL of water through magnetic stirring overnight under ambient conditions. Similarly, chitosan (0.5 and 1 g) was dissolved in acetic acid (10 mL, 100 mM), followed by mild stirring and heating at approximately 55 °C to form a chitosan solution (pH 4). The solutions were left at room temperature for two hours (h) [20].

### 2.3. Sizes, PDI, and Zeta Potential

The size and polydispersity index (PDI) of the generated SLN were measured using a Malvern Zetasizer (Nanotechnology ZS, Zetasizer Ver. 7.13, Malvern, Malvern, UK). The zeta potential of the developed SLNs was determined using a Malvern Zetasizer, and the average of the three readings was calculated [21].

### 2.4. Entrapment Efficiency of SVA (EE %)

The percentage of medication entrapped in the SLNs was calculated by monitoring the drug concentration in the aqueous phase using the ultracentrifugation technique. A specific volume of the prepared SLN (2 mL) was spun to isolate unbound medication or free SVA from the loaded SLN via ultracentrifugation at 3500 rpm for 30 min at 25 °C. The free drug in the supernatant (1 mL) was estimated via diluting it to 10 mL with distilled water as a blank and measuring the absorbance spectrophotometrically at 238 nm. The entrapment efficiency was measured indirectly using the following equation [22]:

(1) $$\mathrm{Entrapment}\;\mathrm{efficiency}\;(\mathrm{EE}\%)=(\frac{W_{i}-W_{f}}{W_{i}})\;\times \;100$$

where (W_i_) is the amount of drug introduced to the technique and (W_f_) is the amount of drug in the supernatant.

### 2.5. SEM

The morphology and surface characteristics of SLNs were examined using the scanning electron microscope (SEM) (Jeol JSM-5400 LV, Jeol, Tokyo, Japan). Prior to examination, the selected SLNs were air-dried under ambient conditions, fixed on a carbon double-adhesive layer that was pre-attached to a specimen stub on a metal holder, and thinly covered with gold using a sputter coater. The SLNs were then scanned at an accelerating voltage of 15 kV.

### 2.6. Viscosity

The viscosity of F6, F11, F6-CS 0.5%, F6-CS 1%, F11-CS 0.5, F11-CS 1%, F6-AL 0.5%, F6-AL 1%, F11-AL 0.5%, and F11-AL 1% were measured using a rotary viscometer (Lamy Rheology, coaxial cylinder measuring system (Tube DIN 3), Champagne-au-Mont-d’Or, France). The rotation speed was between 0.3 and 100 rpm. The shear rates ranged from 0.1 rpm to 100 rpm and 5.1 mm high gaps. The formulations were evaluated at temperatures of ≥25 °C, and the average of three readings was calculated [23].

### 2.7. In Vitro Drug Release Studies

The in vitro drug release of SVA from SLNs was evaluated with two different media. The release study was performed using the dialysis bag diffusion method. A specific volume (2 mL) of each SLN sample, equivalent to 2 mg of SVA, was placed in a dialysis bag, after which it was sunk in 50 mL of the release medium and incubated in a thermally controlled water bath rotating at 100 strokes per min at a temperature of 37 ± 1 °C. During the first 2 h, the pH was maintained at 1.2 pH (0.1 N HCL-sodium lauryl sulfate, 0.1%) using simulated gastric fluid but without enzymes, then the pH was raised to 6.8 for 4, 6, 12, and 24 h using phosphate buffer. The surfactant SLS was used to ensure the release study was conducted under sink conditions. Portions of the release medium (2 mL) were removed at the scheduled times and refilled with fresh release media. The percentage of drugs released was plotted against time (h). The concentration of each withdrawn sample was measured at 238 nm at different times. Data were collected using Lab Solution UV-Vis, Spectronic Shimadzu-UV-1900i^®^ software (version 2.01), Microsoft Excel 2016, and GraphPad Prism.

### 2.8. Release Kinetics

The in vitro drug release results were examined using a linear regression algorithm in accordance with zero-order kinetics, first-order kinetics, the Higuchi diffusion model, the Korsmeyer-Peppas model, the Hixon–Crowell model, and the Baker and Lonsdale model. Release kinetic modeling was used to analyze the mechanism of SVA release from the prepared SLN, using the following equations:

1. Zero-order kinetics:

(2) A=A0−Kt

where (A) is the amount of drug released at the time (t), (A0) is the initial amount of drug in the solution, and (K) is the zero-order release constant [24].

2. First-order kinetics:

(3) logA=logA0−Kt/2.303

where (A) is the amount of drug released at the time (t), (A0) is the initial amount of drug in the solution, and (K) is the first-order release constant [24].

3. Hixson–Crowell model:

(4) $$\sqrt[{3}]{1-f_{i}}=1-K_{HC}\;t$$

where fi = 1 − (W_i_/W_0_) and indicates the part of the drug that was released at time (t), and (K_HC_) is a release constant [25,26].

4. Korsmeyer-Peppas kinetics model:

(5) $$Mt/M\infty ={Kt}^{n}$$

5. Baker and Lonsdale kinetic model:

(6) 321−1−MtM∞23−MtM∞=Kt

where (K) is the release constant at time (t). This equation can be used to linearize the release data for various nanoparticle formulations (microcapsules and microspheres) [26].

6. Higuchi kinetic model:

(7) Q=K√t

where K denotes the Higuchi release constant.

### 2.9. In Vivo Study

The animal study was performed with a university license and authorization, according to the Animal Ethics Committee (AEC) of the Faculty of Pharmacy, Minia University (MPE 230111). Male New Zealand albino rabbits weighing 1.5–2.0 kg were purchased from an animal house. The rabbits were randomly divided into three groups. Each group contained five rabbits. The first group was administered the SVA suspension (10 mg/kg) as the control group. The second group was administered formulation F11. The third group was administered the F11 chitosan 1% formulation (chitosan completed to 10 mL in the aqueous phase) at a dose of 10 mg/kg. Blood samples (2.5 mL) were collected from the marginal ear vein at 0.5, 1, 2, 4, 6, 8, 12, and 24 h after administration and transferred to heparinized tubes. Plasma was isolated using centrifugation at 3000 rpm for 20 min and was preserved at −20 °C until further analysis. The HPLC system (Dionex Ultimate™ 3000 HPLC (Thermo Scientific™, Waltham, MA, USA) HPG-3400SD pump and DAD-3000 (RS) detector) was equipped with a Hypersil Gold C18 (15 × 0.46 cm, 5 µm) column (Phenomenex, Luna, Torrance, CA, USA). A security guard C18 (4 × 3.0 ID) was employed. The mobile phase was composed of acetonitrile–water (65:35 *v*/*v*), pH 3.5, a flow rate of 1.0 mL/min, and an injection volume of 10 μL. A UV detector operating at a wavelength of 238 nm was used. Data were analyzed using WinNonlin^TM^ version 1.5 (Pharsight, Apex, NC, USA) [27]. The relative bioavailability was estimated using Equation (8) as follows:

(8) $$Relative\;bioavilability\;\%=\frac{AUC\;sample}{AUC\;refrence\;drug}\times 100$$

### 2.10. Statistical Analysis

GraphPad Prism version 8.4.3 Software, San Diego, CA, USA, www.graphpad.com (accessed on 2 June 2023), was used to conduct an unpaired *t*-test and a one-way ANOVA followed by a Dunnett’s multiple comparisons test.

## 3. Results and Discussion

Thirteen SLNs were successfully prepared from four lipids (Compritol 888 ATO, Geleol, Precirol ATO 5, and Gelucire 43/01). The melting points reported for these four lipids are 70, 59, 55, and 43 °C, respectively [28]. The HLB values recorded for the four lipids were 1, 3, 2, and 1, respectively. These four lipids are chemically known as glyceryl dibehenate (C_25_), mono- and diglycerides (C_21_), glyceryl palmitostearate (C_37_), and mono-, di-, and triglyceride (C_23_) polyethylene glycol, respectively [29].

**Figure 1:**
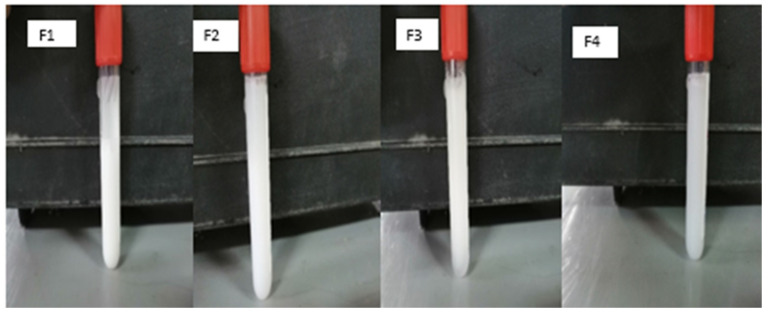
Photographs of the four different bare SLNs with milky dispersion textures.

The two following stabilizers were used: Poloxamer 407 and Tween 80. Two lipid concentrations (200 and 400 mg) were investigated. The four lipids were used separately or in various combinations of two or more lipids to generate SLNs using the hot homogenization technique. This method was successful in the preparation of thirteen different SLNs. The SLN dispersions instantly turned milky white due to the formation of SLNs [30], as shown in Figure 1.

### 3.1. Particle Size and Polydispersity Index

The particle size and polydispersity index (PDI) are critical variables that influence the stability, release rate, and biological performance of SLN systems [31].

The SLNs were in the nanoscale range (157 ± 16.35 nm to 1181 ± 129.7 nm), as shown in Table 2. SLNs can vary in particle size and PDI due to their lipid nature, type of stabilizer, and concentrations of both lipids and stabilizers [32]. The particle sizes recorded for F1–F4 (composed of different lipids with the same total lipid and stabilizer concentrations) were 663.9 ± 90.92 to 1167.67 ± 73.33 nm, respectively. These results were significant (*p* < 0.05). Gelucire 43/01-based SLNs generated the largest sizes; the lowest sizes recorded were with both Precirol- and Geleol-based SLNs, while Compritol 888 ATO-based SLNs were measured in the middle size range.

F4 showed significantly (*p* < 0.05) larger particle sizes than the other three (F1–F3), whereas F2 and F3 exhibited the lowest particle sizes (339 and 333 nm, respectively). F1 was in the middle, with a particle size of 664 nm. Different lipids generate SLNs of different sizes [32]. Both Precirol ATO 5 and Geleol seem to be superior in generating SLNs when compared to Compritol 888 ATO and Gelucire 43/1 [33].

This could explain the larger particle sizes for the F3 formula only. However, it was noticed that F4 with C_23_, F1 with C_25_, and F2 with C_37_ generated SLNs of 1167.67 ± 73.33, 663.9 ± 90.92, and 338.57 ± 52.86 nm, respectively. Table 2 shows that, in formulations in which the hydrocarbon chain length increased, the particle size decreased [34]. A PDI of approximately 0.1 indicated a good uniform distribution.

A lower HLB value for lipids is likely to cause growth in the nucleus and diminish the final particle size [35]. When increasing the lipid concentration (Compritol HD5 ATO) from 200 mg to 400 mg, it leads to a reduction in the particle size from 663.9 nm (F1) to 396.7 nm (F5) (Table 2). The particle size decreased with the increased concentration of lipids (F2, 338.57 nm to F6, 231.87 nm), (F4, 1167.67 nm to F8, 871.83 nm), as shown in Table 2, with (F3, 332.6 nm of F7, 387.3 nm) [15].

When the concentration of lipids with smaller HLB values increases, the nucleus grows more slowly, and the ultimate particle size decreases [35,36]. On the contrary, the results showed that increasing the concentration of Geleol was not associated with a decrease in the particle size [36].

With regard to the polymeric coating of SLNs, particle sizes of F6, F6-CS 0.5%, F6-CS 1%, F6-AL 0.5, and F6-AL 1% increased as follows: 231.87 ± 108.22, 487.5 ± 13.09, 844.4 ± 117.9, 1181 ± 129.7, and 906.0 ± 42.12 nm, as shown in Table 2. Coated SLNs had significantly (*p* < 0.05) smaller particle sizes than bare SLNs, which could be due to the capacity of the hydrophilic polymers to improve the stability of SLNs via steric hindrance and inhibit particle aggregation.

The higher the concentrations of the coating chitosan and alginate polymers, the larger the recorded particle sizes. These increases were significant (*p* < 0.05) and could be attributed to multilayer coatings with alginate sodium of different thicknesses. The main characteristic of these hydrocolloidal polymers could be associated with the increasing hydrodynamic diameters of the coated SLNs due to the gelling properties of sodium alginate in aqueous solutions [37].

Similarly, the particle sizes of F11, F11-AL 0.5%, and F11-AL 1% were 260.1 ± 3.72, 157 ± 16.35, and 1071 ± 45.63 nm, respectively. The particle sizes of F11 chitosan (0.5 and 1%) were 477.3 ± 21.4 and 524.3 ± 80.31 nm, respectively. F11 with an AL coating decreased the particle size, leading to an increase in the outside aqueous phase and the composition of nanostructures that were less likely to aggregate because of the steric hindrance of the hydrophilic chain coating on the SLNs [38]. As the CS concentration increased, the corresponding particle size also increased. The lack of water solubility of chitosan and the bulk of amine groups that were unprotonated and failed to engage in ionic interactions caused an increase in the particle size [39]. The calculated *p*-value (*p* = 0.0002) was significant.

Particle size analysis revealed that the smallest particle size was obtained from F11, approximately 260.1 nm. In contrast, F12, which was prepared with the same method, produced the largest particle size of approximately 894.3 nm (Table 2) [40]. The HLB value is an essential indicator of SLN particle size, as an increased lipophilicity decreases the particle size via reducing the HLB value. Compritol 888 ATO and Precirol ATO5 had lower HLB values, whereas Gelucire 43/01 had high HLB values.

### 3.2. Zeta Potential (ζ) Analysis

The zeta potential measures the magnitude of repulsion between like-charged colloidal systems. During storage, particle aggregation can be partially avoided by repulsive forces. Therefore, zeta potential is a sign of the physical stability of a formulation [41]. All zeta potential values indicated lower range values recorded at ζ-values −21.86, −5.53, −4.92, and −4.79 mV in F3, F2, F1, and F4, respectively (Table 2). The zeta potential analysis of SAV-loaded lipid nanoparticles showed negative ζ-values. This can be ascribed to the anionic and acidic nature of SVA. These results agree with previously reported data [42]. The results indicated that a low zeta potential was observed for SAV-SLNs created from Poloxamer 407 as a non-ionic surfactant, which may be due to the chemical nature of Poloxamer 407, which serves as a steric stabilizer and reduces the zeta potential, owing to the movement in the electric shear plane of particles [43].

Table 2 illustrates that F10 recorded a ζ-value of −2.5 mV. F10 was composed of a combination of equal amounts of Geleol and Compritol 888 ATO. This could be due to an increase in the surface coverage of SLNs, which minimized the electrophoretic mobility and lowered the zeta potential while increasing the hydrocarbon chain of lipids, which induced crowding in the media. Increasing the HLB content of the lipid mixture reduced the zeta potential [44,45]. Therefore, polymeric coatings were studied to increase the viscosity, form structured vehicles, and impart steric stabilization [46]. Furthermore, the anionic, and cationic coating polymers of alginate and chitosan were used to increase the magnitude of the zeta potential of bare SLNs. The observed results showed ζ-values of 44.7 mV, −22.0 mV, 33.4 mV, and −9.48 mV for the F6 chitosan, F6-AL, F11-CS, and F11-AL, respectively, as shown in Table 2. The observed results showed ζ-values of 44.7 mV, 52.9 mV, −22.0 mV, and −27 mV for F6-CS 0.5%, F6-CS 1%, F6-AL 0.5%, and F6-AL 1%, respectively.

Adding chitosan coating caused the surface charge to be inverted to a positive charge. SLNs were prepared using Na-AL, an anionic linear polysaccharide. They produced negative zeta potential values because of free [COO^−^] [47,48]. All zeta potential values recorded for the coated SLNs indicated the good stability of the SLN-coating system, with ζ-values above +/− 30 mV, which required at least 30 mV to produce a stable SLN system [49,50,51]. When the concentration of the coating polymers was increased, the ζ-value increased [51,52]. Because of the increased surface coverage of the SLNs, the amine group and [COO¯] concentrations increased. The increased zeta potential led to an enhanced physical stability and prevented sedimentation during storage.

### 3.3. Entrapment Efficiency (EE%)

All SLNs exhibited a high entrapment efficiency of almost 100%, as shown in Table 2. Comparable results were observed elsewhere for lipophilic drugs, such as simvastatin; the log P value of simvastatin was 4.3. Hence, simvastatin is considered an extremely lipophilic drug [53]. The higher the concentrations of lipid-forming SLNs used, the greater the EE%, especially for F5, F6, F7, and F8, in which the lipid concentrations increased from 200 mg to 400 mg. The recorded entrapment efficiency was the complete encapsulation of the lipophilic drug at 101.01%, 100%, 99.26%, and 99.26% (Table 2).

### 3.4. Scanning Electron Microscope (SEM)

**Figure 2:**
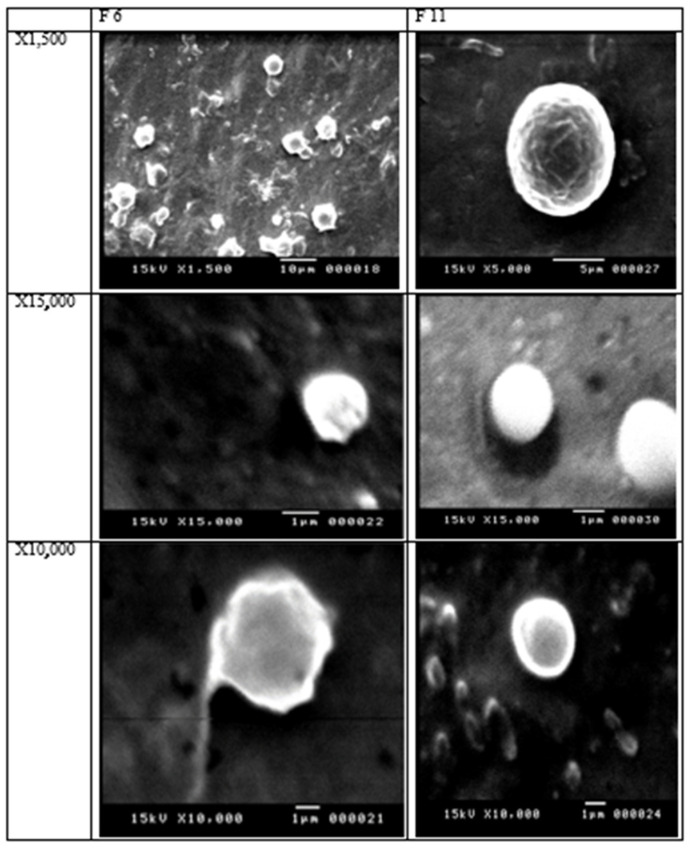
Representative SEM micrographs of F6 and F11 SLNs at different magnifications.

SEM images of the selected SLNs (F6 and F11) demonstrated the smallest particle sizes and a large percentage of entrapment efficiency. The SEM images of the two SLNs showed spherical and smooth particle surfaces. F6 and F11 were prepared using 400 mg Precirol and a mixture of the three lipids, Compritol 888 ATO, Precirol, and Geleol (1:1:1) *w*/*v*. The scanning electron microscopy of SLNs F6 and F11 showed spherical morphology and smooth surfaces. Free drug crystals were not observed. Figure 2 shows the SEM images of the prepared batches.

### 3.5. Rheological Studies

The surface coating of SLNs offers superior advantages over bare SLNs. Coated SLNs are likely to show improved muco-adhesive characteristics, a prolonged release pattern, and better drug delivery effectiveness [54,55]. Coated SLNs behave similarly to shear-thinning gels, which act like liquids under stress, but change to a viscous form when the stress is relaxed. Shear thinning is a term used to describe this type of behavior under pressure [23,56].

#### 3.5.1. Effect of Lipids on Viscosity

**Figure 3:**
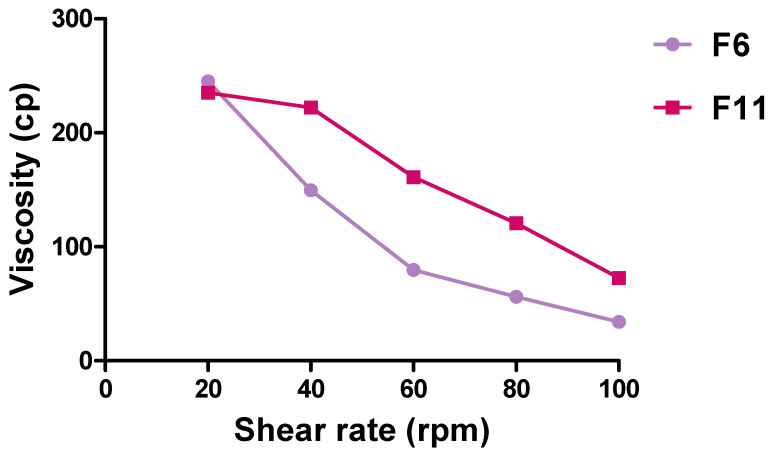
Rheological profiles (viscosity (cp) versus shear rates (rpm) for selected bare SLNs; Data points represent means; n = 3.

Figure 3 shows that F11 (161 cp) had a two-fold higher viscosity than F6 (79.66 cp) at 60 rpm. The rheological behavior of the two SLNs was pseudo-plastic (shear thinning). The viscosity of SLNs is expected to decrease with increasing shear rates.

For example, F11 at shear rates of 20 rpm and 40 rpm was 235 cp and 222 cp, respectively. This type of behavior under stress conditions is called shear thinning [46].

#### 3.5.2. Effect of Polymer Concentrations on Viscosity

**Figure 4:**
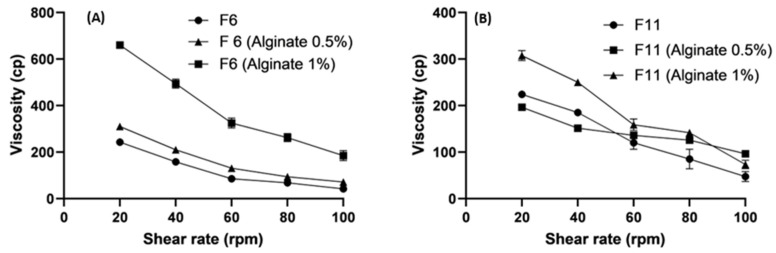
Rheological profiles (viscosity vs. shear rates) of bare and alginate coated SLNs; (**A**) and (**B**) were bare and alginate coated F6 and F11; respectively. Data points represent means ± SD, n = 3.

**Figure 5:**
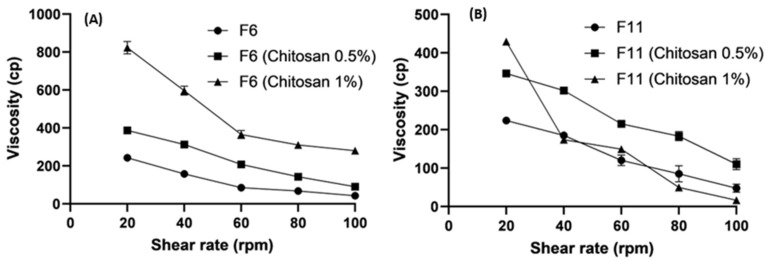
Rheological profiles (viscosity vs. shear rates) of bare and chitosan-coated SLNs; (**A**,**B**) were bare and chitosan-coated F6 and F11, respectively. Data points represent means ± SD, n = 3.

The formulations (F6 and F11) changed significantly when the concentrations of polysaccharides such as chitosan and alginate increased from 0.50% to 1.0%. Figure 4 and Figure 5 demonstrate the increased viscosity of the system due to the higher concentrations of polysaccharides. Figure 5 shows an abrupt decrease in the viscosity of F11 (Chitosan 1%) because of the increase in thew lipid content and the high concentration of chitosan. Shear-thinning gels exhibit pH-responsive behavior [57,58].

The high viscosity provided beneficial information regarding releasing the active ingredient from the coated SLNs. The drug release is more controlled in the highly viscous environments, which affects the bioavailability and therapeutic efficacy [59]. The results related to viscosity can also reveal the resistance of a composition to structural breakdown. They also affect the function and stability of a product [60].

### 3.6. In Vitro Drug Release

**Figure 6:**
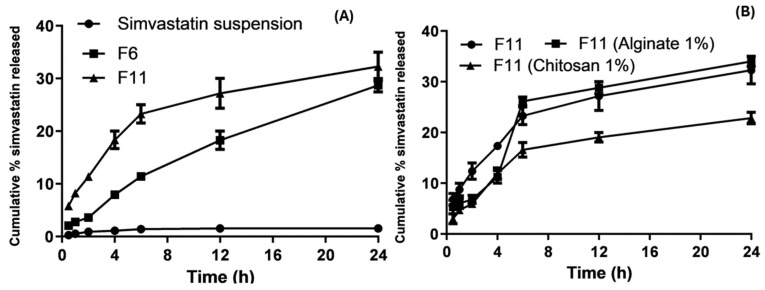
In vitro drug release profile of simvastatin suspension, bare F6 and F11 (**A**); and bare F11, coated F11 with alginate (1%) and chitosan (1%) (**B**) at pH 1.2 and pH 6.8. Data points represent means, n = 3.

The in vitro release of the SVA suspension resulted in slow and low cumulative release rates. After 6 h and 24 h at pH 6.8, it was found to be unchanged at 1.35% and 1.4397%, respectively, as shown in Figure 6. The cumulative percentage of SVA SLNs from F6 and F11 after 24 h was 27.5 and 29.6%, respectively. This significant (*p* < 0.01) enhancement in the in vitro release rates of poorly soluble drugs could not be achieved without formulating SVA into SLNs. The molecular dispersion of the drug in the lipid and decreased PS of 260.1 nm increased the surface area and solubility of the drug, thereby improving the drug dissolution [61]. Moreover, there was a significant difference (*p* < 0.05) in the drug release between formulations F6 and F11. Figure 6 shows that F11 chitosan 1% had a low cumulative percentage of SVA release of 15.6% after 24 h because the high concentration of lipids and coating with a high chitosan concentration led to an increase in viscosity.

Several variables may affect how quickly the medication is released from the SLNs. According to one report, a quick release is facilitated by a number of characteristics, including a wide surface area, a high diffusion coefficient resulting from a small molecular size, minimal medium viscosity, and a small drug diffusion length. A slow drug release is possible when the drug is homogeneously distributed in a lipid mixture [62]. Finally, F11 had a higher cumulative percentage of SVA release than the coated SLNs.

#### Release Kinetics Design for Optimized SLNs and Coated Nanoparticles

The kinetics of drug release were studied to elucidate the mechanisms of drug release. The zero-order, first-order Baker and Lonsdale model, Hixson–Crowell model, Higuchi model, and Korsmeyer model were employed to fit in vitro release data. The best model was selected based on the regression square root (R^2^). The exponent of the Korsmeyer equation (n) was used to evaluate the release mechanism of SLNs. Table 3 shows that the Higuchi model is the best-fit model to illustrate the release mechanism. The Higuchi model was chosen based on the goodness of fit parameters of the highest regression root square (R^2^) of 0.9846 and the smallest mean rate constant (KHC) of −4.17, which resulted in a prolonged cumulative percentage drug release of SVA for F6. F11 was best fitted by the Korysmeyer-Peppas model because it had the highest regression root square (R^2^) of 0.97 and n = 0.45, which indicated a Fickian diffusion-mediated release. The values of the correlation coefficient (R^2^) for all formulations were high for formulations F6-AL 0.5%, F6 (Chitosan 1%), F11 (Chitosan 1%), and F6 (Chitosan 0.5%) to evaluate the drug release behavior. However, the most significant contribution of coupled diffusion erosion release and polymer relaxation (non-Fickian) was noticed at n values of 0.5 < n < 1.0 (Table 3). F6 (Chitosan 1%) was best described by the Higuchi model because it had the highest regression root square (R^2^) of 0.92 and a low-rate constant (K), indicating the sustained release of the SVA drug (*p* < 0.0001, one-way ANOVA). F11 (Chitosan 1%) was fitted by the Korysmeyer-Peppas model because the highest regression root square (R^2^) of 0.95 and n = 0.47 indicated a Fickian diffusion-mediated release.

### 3.7. In Vivo Pharmacokinetic Evaluation

**Figure 7:**
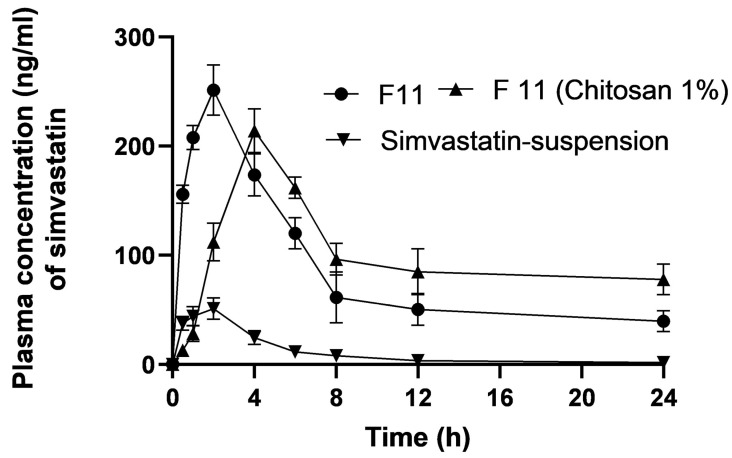
Plasma concentrations versus time curve for simvastatin suspension (the control for a conventional release system), bare SLNs (F11), and coated F11 (chitosan 1%). Data points represent means ± SD, n = 3.

The mean plasma concentration versus time profiles of the drug suspension (Group 1), bare F11 (Group 2), and coated F11 chitosan 1% (Group 3) are shown in Figure 7, and their corresponding pharmacokinetic parameters are shown in Table 4. Following the oral administration of the SVA suspension (as a control sample) (10 mg/kg), the C_max_ was 57.17 ng/mL. Similar plasma levels of simvastatin C_max_ 47.72 ng/mL) after oral administration have been reported in a previous study [63]. Due to the increased esterase enzyme concentration in rabbits, SVA was immediately transformed into its metabolite (exposed first-pass metabolism), which may explain the low bioavailability of SVA alone [63]. The C_max_ values following the oral administration of 1% bare F11 and coated F11 chitosan were 251.88 and 228.3 (ng/mL), respectively. The C_max_ of F11 and chitosan-coated F11 showed a statistically significant (*p* < 0.05) increase of 4.4 times and 4 times, respectively. Additionally, the relative bioavailability when compared to the SVA suspension for bare F11 and coated F11 chitosan 1% was calculated to be 697% and 1309.66%, respectively.

Formulations may have enhanced the bioavailability due to their near 260.87 ± 108.22 nm particle size, large surface area, and accumulative % release of SVA-SLNs. These factors also enhance the bioavailability through increasing C_max_ and extending the retention time at the absorption site. The F11-SLNs formulation contained Poloxamer 407, which could reduce enzymatic activity to extend its stability against hepatic enzymatic degradation. The impact of SLNs on stability, mucoadhesion, and cellular absorption was examined by Luo et al. [27,64]. SLNs coated with chitosan (F11 chitosan, 1%) showed a sustained drug release of simvastatin to prevent rapid conversion into its metabolite and to enable the measurement of exact pharmacokinetic parameters [65]. Two parameters are likely to affect the plasma concentrations from coated SLNs as follows: firstly, when the concentration of chitosan is increased, the protective effect against physical stress decreased and the particle size is increased to 844.4 ± 117.9 nm, and an increase in the diameter index leads to a decrease in the surface area [66]. As a result, the SLNs outer layer was sufficiently covered with chitosan, but not too much to cause “depletion flocculation”, and imparted steric stabilization [67,68]. Secondly, the pH effect on the stabilization of chitosan with a high concentration revealed that the chitosan is positively charged and more soluble at a low pH, resulting in an increase in the release rate of SVA when exposed to the acidic gastric environment and vice versa in the intestinal pH. Therefore, a noticed decrease in the plasma concentration of SVA after 2h was shown from F11 chitosan 1% (Figure 7). Chitosan becomes insoluble at high pH [67]. The peak plasma concentration of SVA from the coated SLNs remained well below that for uncoated SLNs. Nevertheless, further higher plasma concentrations were recorded for the coated SLNs (Figure 7). Due to the free amino groups of chitosan, this allowed the formation of hydrogen bonds with the hydroxyl groups of the mucosal glycoproteins, increasing mucoadhesiveness and enhancing absorption further [69].

The T_max_ value following the oral administration of the SVA suspension was 1.41 h, while the T_max_ values for F11 and F11 chitosan 1% were 1.74 and 5.15 h, respectively. The coated F11 showed a more sustained drug release, and these results agree with the release data. The AUC_0→24_ increased from 272.91 ng·h/mL for the SVA suspension to 1880.4 and 3562.18 with 1% F11 and F11 chitosan, respectively, after the administration of a single oral dose (10 mg). As shown in Table 4, the modified release SLNs showed an extended mean residence time (MRT) when compared to the immediate release from the SVA suspension. The mean residence times (MRT) of the SVA suspension, F11, and F11 chitosan 1% formulations were 3.7, 6.29, and 19.1 h, respectively. This showed that the F11 and F11 chitosan 1% formulations had a stronger retardation effect than the quick-release SVA medication. According to the study findings, the primary problem with the oral absorption of statins, particularly simvastatin, is that it is eliminated by hepatic first-pass metabolism. SLNs offer opportunities for transcellular, paracellular, and lymphatic transport. These are the three primary processes hypothesized for the absorption of SLNs from the gut [64,70] as follows:

1. Enzymatic lipid breakdown in the small intestine results in the formation of monoglycerides and diglycerides, which eventually separate from the surface of nanoparticles and form micelles. These micelles combine with surface-active bile salts to generate mixed micelles, which are then absorbed via a transcellular pathway.
2. Chylomicrons created from micelles eventually reach the lymphatic system. These micellar products could improve the in vivo drug dissolution rates and the ability of enterocytes to absorb them.
3. Lipid nanoparticles (SLNs) can penetrate M cells via transcytosis, travel to the lymphatic system, and exit the systemic circulation through the thoracic duct. They have particular benefits in lymphatic transport due to chylomicron production, rapid absorption by M cells, and improved bioavailability by extending their time at the absorption site and preserving the medication from hepatic breakdown and the potential intestinal wall.

## 4. Conclusions

Uncoated (bare) and coated lipid nanoparticles were prepared with different lipid concentrations. The coats were composed of anionic and cationic hydrophilic polymers. The most effective formulation achieved the best characterization, particle size, zeta potential, and in vitro drug release properties. The nano-formulations exhibited the best rheological behavior (shear-thinning behavior). Ex vivo and in vivo studies have shown that synthesized SLNs contain particles in the nanoscale range. F11 was the optimized SLN due to its small particle size (260.1 ± 3.72 nm), higher entrapment efficiency, and higher accumulative SVA release (29.6%). Bare F11 and coated F11 chitosan 1% had higher relative bioavailability (691.28% and 1309.66%, respectively). However, bare F11 demonstrated superior absorption rates and total bioavailability when compared to the coated systems. The smaller particle size (nanoscale) and increased surface area paired with the sustained release of F11 and F11 (Chitosan 1%) were contributing factors to improve the bioavailability of SVA. Additionally, improving the lymphatic transport and preserving the medication from hepatic breakdown and potential intestinal wall during absorption help to increase the oral bioavailability of chitosan-coated SLNs. Future research should explore the potential of using SLNs to improve and maintain the oral bioavailability of statins and other drugs with low solubility and extensive hepatic first-pass metabolism.

## Figures and Tables

**Table 1:** Composition of the prepared SLNs.

	F1	F2	F3	F4	F5	F6	F7	F8	F9	F10	F11	F12	F13
Compritol 888 ATO (mg)	200				400				200	200	100		100
Precirol (mg)		200				400			200		100	100	100
Geleol (mg)			200				400			200	100	100	100
Gelucire43/01 (mg)				200				400				100	100
Poloxamer407 (mg)	100	100	100	100	100	100	100	100	100	100	100	100	100
Tween 80 (%)	0.5	0.5	0.5	0.5	0.5	0.5	0.5	0.5	0.5	0.5	0.5	0.5	0.5

**Table 2:** Particle size, polydispersity index, zeta potential, and% EE of bare SLNs and coated SLNS; Data represent means ± SD, n = 3. * Denotes a statistically significant difference; ** denotes a statistically non-significant difference.

Formulation Code	P.S (nm)	PDI	ζ (mV)	% EE
F1	663.9 ± 90.92	0.612 ± 0.084	−4.92 ± 0.22 **	100 ± 2.85 **
F2	338.57± 52.86 **	0.438 ± 0.051	−5.53 ± 0.49 **	99.75 ± 0.52
F3	332.6 ± 7.71 **	0.907 ± 0.03	−21.86 ± 1.52 *	97.97 ± 0.39
F4	1167.67 ± 73.33 *	0.611 ± 0.09	−4.79 ± 0.13	98.96 ± 2.66
F5	396.7 ± 8.63	0.832 ± 0.073	−3.21 ± 0.15	101.01 ± 2.85
F6 *	231.87 ± 108.22	0.4926 ± 0.014	−1.8 ± 0.15	100.1 ± 2.55
F7	387.3 ± 1.7 **	0.518 ± 0.013	−6.75 ± 1.7	99.26± 1.56
F8	871.83 ± 26.25	0.65 ± 0.034	−2.57 ± 0.23	99.26 ± 0.28
F9	765.17 ± 29.18	0.619 ± 0.002	−8.17 ± 0.63	99.72 ± 0.41
F10	528.27 ± 20.88	0.772 ± 0.12	−2.5 ± 0.39	99.20 ± 0.32
F11 *	260.1 ± 3.72	0.409 ± 0.005	−4.26 ± 0.26	99.36 ± 0.68
F12	894.3 ± 56.14	0.611 ± 0.034	−7.97 ± 0.71	99.44 ± 1.21
F13	488.4 ± 19.24	0.738 ± 0.11	−7.98 ± 0.81	99.53 ± 0.28
F6 (Chitosan 0.5%)	487.5 ± 13.09 *	0.127 ± 0.028	44.7 ± 1.63	98.999 ± 0.02
F6 (Chitosan 1%)	844.4 ± 117.9 *	0.504 ± 0.057	52.9 ± 2.24	98 ± 0.28
F6 (Alginate 0.5%)	1181 ± 129.7 **	0.831 ± 0.072	−22.0 ± 0.781	97.9866 ± 0.23
F6 (Alginate 1%)	906.0 ± 42.12 **	0.621 ± 0.09	−27 ± 0.321	97.99 ± 0.06
F11 (Chitosan 0.5%)	477.3 ± 21.4	0.504 ± 0.057	33.4 ± 1.85	99.4793 ± 0.08
F11 (Chitosan 1%)	524.3 ± 80.31	0.249 ± 0.016	42.6 ± 1.75	99.756 ± 0.15
F11 (alginate 0.5%)	157 ± 16.35	0.067 ± 0.019	−9.48 ± 0.174	98.798 ± 0.10
F11 (Alginate 1%)	1071 ± 45.63	0.852 ± 0.084	−24.8 ± 0.346	99.314 ± 0.31

* F6 and F11 were selected for polymeric coating and further investigations. The two selected formulations showed a nano-sized range (230 and 260 nm) and were composed using a single or a combination of the three lipids.

**Table 3:** Release kinetics models for F6, F11 and coated-SLNs.

Code Formula	Zero		First		Higuchi		Hixon–Crowell		Baker & Lonsdale		Korysmeyer-Peppas	
	R^2^ *	K **	R^2^	K	R^2^	K_H_	R^2^	K_HC_	R^2^	K_3_	R^2^	N ***
SVA	0.58	0.72	0.49	−0.16	0.76	0.44	0.52	0.01	0.63	0.001	0.88	0.33
F6	0.98	2.29	0.79	0.50	0.98	−4.17	0.87	0.07	0.97	−0.001	0.97	0.72
F11	0.89	9.8	0.64	2.23	0.85	2.02	0.69	2.1	0.92	0.001	0.97	0.45
F6 (Alginate 0.5%)	0.81	7.81	0.79	0.879	0.92	−0.21	0.74	0.006	0.91	0.0006	0.92	0.53
F6 (Alginate 1%)	0.738	15.81	0.665	1.187	0.88	8.27	0.69	0.04	0.81	0.005	0.93	0.33
F6 (Chitosan 0.5%)	0.69	25.12	0.54	1.3	0.86	1.33	0.69	0.09	0.96	−0.006	0.87	0.61
F6 (Chitosan 1%)	0.775	3.22	0.58	0.397	0.92	−1.11	0.65	0.058	0.91	0.0002	0.94	0.79
F11 (Alginate 0.5%)	0.75	10.79	0.67	1.008	0.86	0.55	0.7	0.065	0.83	0.002	0.89	0.51
F11 (Alginate 1%)	0.79	8.77	0.7	0.93	0.89	0.896	0.73	0.06	0.88	0.001	0.89	0.48
F11 (Chitosan 0.5%)	0.68	32.13	0.51	1.4	0.85	9.97	0.57	0.075	0.90	0.02	0.92	0.53
F11 (Chitosan 1%)	0.83	6.30	0.68	0.78	0.95	1.27	0.73	0.05	0.93	0.005	0.95	0.47

* (R^2^) is regression root square and measure closeness of the data; ** (K) Means rate constant; *** (N) Related to Korsmeyer-Peppas release exponent.

**Table 4:** Mean pharmacokinetic parameters ±SD the after oral administration of the SVA suspension, F 11, and F 11 chitosan 1%. Data represent means ± SD, n = 3.

Parameters	SVA Suspension	F11	F11 (Chitosan 1%)
AUC_0→24_ (ng·h/mL) *	272 ± 197.89	1880.4 ± 222.08	3562.18 ± 1256.03
C_max_ (ng/mL) **	57.1.6 ± 22.93	251.88 ± 17.50	228.3 ± 21.76
T_max_ (h) ***	1.41 ± 0.32	1.74 ± 0.36	5.15 ± 1.13
t_1/2_ (h) ****	0.54 ± 1.12	3.97 ± 0.99	11.43 ± 5.85
MRT (h) *****	3.7 ± 0.52	6.29 ± 0.84	19.1 ± 1.03
K_a_ (h^−1^)	0.604 ± 0.06	1.32 ± 0.21	0.40 ± 0.03
K_el_ (h^−1^)	0.160 ± 0.02	0.030 ± 0.02	0.011 ± 0.05
t1/2Ka (h)	1.15 ± 0.12	0.52 ± 0.04	1.52 ± 0.20
F%		691.28 ± 0.61	1309.66 ± 0.92

* AUC (0→24) = Area under the concentrations versus time curve from time zero extrapolated to 24; ** C_max_ = maximum plasma concentration; *** T_max_ = Time of occurrence of C_max_; **** t_1/2_ = Terminal phase half-life; ***** MRT = mean retention time; and F% = relative bioavailability.

## Data Availability

The data can be shared upon request.
